# Targeting ferroptosis: A novel therapeutic strategy for the treatment of mitochondrial disease-related epilepsy

**DOI:** 10.1371/journal.pone.0214250

**Published:** 2019-03-28

**Authors:** Amanda H. Kahn-Kirby, Akiko Amagata, Celine I. Maeder, Janet J. Mei, Steve Sideris, Yuko Kosaka, Andrew Hinman, Stephanie A. Malone, Joel J. Bruegger, Leslie Wang, Virna Kim, William D. Shrader, Kevin G. Hoff, Joey C. Latham, Euan A. Ashley, Matthew T. Wheeler, Enrico Bertini, Rosalba Carrozzo, Diego Martinelli, Carlo Dionisi-Vici, Kimberly A. Chapman, Gregory M. Enns, William Gahl, Lynne Wolfe, Russell P. Saneto, Simon C. Johnson, Jeffrey K. Trimmer, Matthew B. Klein, Charles R. Holst

**Affiliations:** 1 BioElectron Technology Corporation, Mountain View, California, United States of America; 2 Stanford Center for Undiagnosed Diseases, Stanford University School of Medicine, Stanford, California, United States of America; 3 Unit of Neuromuscular and Neurodegenerative Disorders, Bambino Gesù Children’s Research Hospital, Rome, Italy; 4 Clinical Division and Research Unit of Metabolic Diseases, Bambino Gesù Children's Hospital, Rome, Italy; 5 Children’s National Rare Disease Institute, Children's National Health System, Washington, D.C., United States of America; 6 Department of Pediatrics, Division of Medical Genetics, Stanford University School of Medicine, Stanford, California, United States of America; 7 NIH Undiagnosed Diseases Program, National Human Genome Research Institute (NHGRI), National Institutes of Health, Bethesda, Maryland, United States of America; 8 Division of Pediatric Neurology, Department of Neurology, Neuroscience Institute, Seattle Children's Hospital, Seattle, Washington, United States of America; 9 Center for Integrative Brain Research, Seattle Children’s Research Institute, Seattle, Washington, United States of America; 10 Department of Neurology, University of Washington, Seattle, Washington, United States of America; University of Modena and Reggio Emilia, ITALY

## Abstract

**Background:**

Mitochondrial disease is a family of genetic disorders characterized by defects in the generation and regulation of energy. Epilepsy is a common symptom of mitochondrial disease, and in the vast majority of cases, refractory to commonly used antiepileptic drugs. Ferroptosis is a recently-described form of iron- and lipid-dependent regulated cell death associated with glutathione depletion and production of lipid peroxides by lipoxygenase enzymes. Activation of the ferroptosis pathway has been implicated in a growing number of disorders, including epilepsy. Given that ferroptosis is regulated by balancing the activities of glutathione peroxidase-4 (GPX4) and 15-lipoxygenase (15-LO), targeting these enzymes may provide a rational therapeutic strategy to modulate seizure. The clinical-stage therapeutic vatiquinone (EPI-743, α-tocotrienol quinone) was reported to reduce seizure frequency and associated morbidity in children with the mitochondrial disorder pontocerebellar hypoplasia type 6. We sought to elucidate the molecular mechanism of EPI-743 and explore the potential of targeting 15-LO to treat additional mitochondrial disease-associated epilepsies.

**Methods:**

Primary fibroblasts and B-lymphocytes derived from patients with mitochondrial disease-associated epilepsy were cultured under standardized conditions. Ferroptosis was induced by treatment with the irreversible GPX4 inhibitor RSL3 or a combination of pharmacological glutathione depletion and excess iron. EPI-743 was co-administered and endpoints, including cell viability and 15-LO-dependent lipid oxidation, were measured.

**Results:**

EPI-743 potently prevented ferroptosis in patient cells representing five distinct pediatric disease syndromes with associated epilepsy. Cytoprotection was preceded by a dose-dependent decrease in general lipid oxidation and the specific 15-LO product 15-hydroxyeicosatetraenoic acid (15-HETE).

**Conclusions:**

These findings support the continued clinical evaluation of EPI-743 as a therapeutic agent for PCH6 and other mitochondrial diseases with associated epilepsy.

## Introduction

Mitochondrial disease arises through defects in over 150 distinct mitochondrial- or nuclear-encoded genes, but shares a common biochemical signature of cellular energy dysregulation [1]. Defects in genes affecting mitochondrial proteins often result in oxidative stress, electron transport chain (ETC) deficits, and subsequent mtDNA damage. Owing to the central role that mitochondria play in metabolism, clinical manifestations of mitochondrial disease frequently feature severe neurological and neuromuscular dysfunction. One of the most common neurological manifestations of mitochondrial disease is epilepsy, affecting an estimated 35–60% of mitochondrial disease patients [2,3]. The majority of these seizures are reported to be refractory to current antiepileptic therapies [1,4]. Epilepsy associated with inherited mitochondrial disease is thus a serious unmet clinical need requiring new therapeutic approaches that more precisely target underlying disease mechanisms.

Ferroptosis is a recently-described form of iron- and lipid-mediated programmed cell death distinct from apoptosis and necrosis [5] that has been implicated in a growing number of central nervous system disease pathologies [6]. Also referred to as oxytosis or oxidative glutamate toxicity [7,8], ferroptosis can be induced in cells by glutathione (GSH) depletion, cystine-glutamate transporter inhibition, excess glutamate, genetic manipulation of glutathione peroxidase 4 (*GPX4*), or chemical inhibition of GPX4 with small molecules such as RSL3 [9–13]. Biochemical markers of ferroptosis include elevated oxidized polyunsaturated fatty acids (PUFAs) and decreased GSH levels. Ferroptosis can be mitigated via several approaches, including 15-lipoxygenase inhibition, GSH augmentation, iron chelation, and lipid radical scavenging [9–11,14–18].

GPX4 is central to the regulation of ferroptosis as it constitutively reduces PUFA-derived lipid hydroperoxides to their hydroxyl forms in a GSH-dependent reaction. When GSH is limited or GPX4 is inhibited, lipid hydroperoxides, particularly those generated by 15-LO in complex with PEBP1 [19], accumulate over time. This leads to a feed-forward cascade in which lipid hydroperoxides further activate 15-LO and additional lipoxygenase enzymes, committing cells to ferroptosis. Conversely, when the peroxidation of lipids by lipoxygenases is inhibited genetically or pharmacologically, cells are protected from ferroptosis [9,11,15–17].

Genetic suppression of *Gpx4* activity in mice and humans results in seizure phenotypes. Whereas constitutive *Gpx4*-deficient mice demonstrate early embryonic lethality, conditional neuron-specific ablation of *Gpx4* results in viable animals that develop early-onset seizures accompanied by astrogliosis and a loss of hippocampal parvalbumin-positive (PV+) inhibitory interneurons [9]. Seizures, hyperexcitability, and loss of PV+ interneurons were also described in a mouse strain in which the selenocysteine residue in the active site of *Gpx4* was genetically substituted with cysteine, resulting in a constitutive substantial loss of GPX4 catalytic activity [12]. The ultrarare pediatric syndrome Sedaghatian-type spondylometaphyseal dysplasia (OMIM #250220) results from mutations in the *GPX4* gene. Patients with this syndrome present with severe neurological defects including seizures and cerebellar hypoplasia [20–22].

The dystonia, early-onset seizures, astrogliosis, and neurodevelopmental phenotypes of *Gpx4* reduction-of-function mouse ferroptosis models are reminiscent of certain inherited mitochondrial disease-associated epilepsy syndromes [23–25], raising the possibility that ferroptosis contributes to the pathology of these diseases. The presence of increased lipid peroxidation biomarkers in epilepsy patients further suggests that ferroptosis represents a cellular mechanism underlying excitotoxic neuronal injury in seizure disorders [26,27]. Accordingly, targeting either 15-LO with the intent to decrease the formation of lipid peroxides or GPX4 to increase the catalytic clearance of lipid hydroperoxides represent rational therapeutic approaches to modulate ferroptosis as an intervention for mitochondrial disease-associated epilepsy.

Pontocerebellar hypoplasia type 6 (PCH6, OMIM #611523) is a mitochondrial encephalopathic epilepsy disease characterized by intractable seizures [24,28–38] and is caused by mutations in the mitochondrial arginyl-tRNA synthetase *RARS2*. A clinical study reported a reduction in seizure frequency and seizure-related disease morbidity in a small cohort of PCH6 patients treated with the experimental drug EPI-743 [39]. We previously reported that EPI-743 (α-tocotrienol quinone) potently prevents ferroptosis of immortalized mouse striatal cells, perhaps due to observed direct 15-LO enzyme inhibition by its hydroquinone metabolite [17]. These *in vitro* results raised the hypothesis that anti-ferroptotic activity via 15-LO inhibition underlies the observed clinical benefit of EPI-743. We explored this possibility more fully in primary fibroblasts and B-lymphocytes derived from pediatric patients affected by PCH6 and other inherited mitochondrial epilepsy disorders. EPI-743 potently and completely prevented ferroptosis in cells from patients with pediatric epilepsy, consistent with the model that targeting 15-LO, a key enzymatic governor of ferroptosis, may provide a rational therapeutic approach to treat diseases characterized by energetic dysfunction and refractory epilepsy.

## Materials and methods

### Human cells

All cells studied [PCH6 patient fibroblasts (n = 3), Leigh syndrome patient fibroblasts (n = 3), Alpers syndrome patient fibroblasts (n = 1), Rett syndrome patient fibroblasts (n = 1), epileptic encephalopathy, early infantile, 2 patient B-lymphocytes (n = 1), and pediatric apparently healthy donor fibroblasts (n = 7, ages 1d-13y, average 4y)] were obtained with prior written, informed consent. All samples were collected under oversight of local Institutional Review Boards (IRB), as described below. The following cell lines were obtained from the NIGMS Human Genetic Cell Repository at the Coriell Institute for Medical Research: GM01503, GM03672, GM17567, GM23710B, GM01651, GM21811, GM22143, GM22168, GM00969, GM05565, and GM00038. One patient was enrolled in the NIH Undiagnosed Diseases Program [40–43] under protocol 76-HG-0238, “Diagnosis and Treatment of Patients with Inborn Errors of Metabolism or Other Genetic Disorders” (NCT00369421), approved by the National Human Genome Research Institute (NHGRI) IRB. Written informed consent was obtained from the patient’s parents. The remaining patient-derived cells were obtained under the oversight and approval of the Stanford University IRB, the Children’s National Rare Disease Institute—Children's National Health System IRB, and the Seattle Children's Hospital IRB. In all instances where patient cells were provided, written, informed consent was obtained from the patient’s parents.

### Cell culture

Unless otherwise specified, cells were cultured in high-glucose (25 mM) DMEM medium (Gibco) supplemented with fetal bovine serum (FBS, 10% v/v; Sigma), penicillin (100 U/mL; Gibco), and streptomycin (100 μg/mL; Gibco). Subject 4 Leigh Syndrome fibroblasts were cultured in MEM (Gibco), FBS (25% v/v, Sigma), 1 mM sodium pyruvate (Gibco), penicillin (100 U/mL; Gibco), and streptomycin (100 μg/mL; Gibco). For routine maintenance, fibroblast cells were passaged every 3–4 days, maintaining sub-confluent cell densities. For FeC-BSO ferroptotic challenge studies, fibroblasts were cultured in MEMα medium (Gibco) supplemented with FBS (10% v/v; Corning or Sigma), penicillin (100 U/mL; Gibco), and streptomycin (100 μg/mL; Gibco) and passaged every 4–5 days, maintaining sub-confluent cell densities.

B-lymphocytes were cultured in RPMI-1640 medium (Gibco) supplemented with FBS (10–15% v/v; Sigma), penicillin (100 U/mL; Gibco), and streptomycin (100 μg/mL; Gibco). For routine maintenance, B-lymphocytes were passaged every 3–4 days, maintaining densities under 1 million cells per mL.

### RSL3 cell survival assays

Ferroptotic cell death was induced in patient-derived fibroblast cells using the GPX4 inhibitor RSL3 [44]. Fibroblasts were resuspended in assay medium (DMEM + 10% FBS + penicillin/streptomycin) and seeded at 500 cells per well in clear bottom, black wall, 384 well, tissue culture-treated polystyrene microplates (Corning) using either an electronic multichannel pipette or a Multidrop Combi Reagent Dispenser (ThermoFisher Scientific). Cells were incubated for 18 h at 37°C (95% humidity, 5% CO_2_) to allow attachment. A D300e Digital Dispenser (Tecan) or manual pipetting was used to administer test compounds to the desired final concentrations, followed within 15 min by RSL3 (2 μM final concentration). DMSO diluent was back-filled to a final concentration of 0.3% (v/v). Cell plates were incubated for 24 h at 37°C (95% humidity, 5% CO_2_). After a 15 min equilibration to room temperature, cell viability was assessed using CellTiter-Glo 2.0 (Promega), which was added using the Multidrop Combi Reagent Dispenser (Thermo). After 15 min of incubation at room temperature in the dark, the luminescence per well was determined on a Synergy plate reader (BioTek, 100 ms integration time). Data were imported into and analyzed using Dotmatics Studies software suite. EC_50_ values were estimated using standard four-parameter curve fitting algorithms. EPI-743 treatment typically restored viability to levels comparable with untreated cells, indicating complete rescue.

To assess RSL3-mediated ferroptotic cell death in B-lymphocytes using RSL3, cells were seeded at 40,000 cells per well in 96 well tissue culture-treated polystyrene plates (Falcon) using an electronic multichannel pipette. EPI-743 and RSL3 were administered to the desired final concentrations using a D300e Digital Dispenser (Tecan). DMSO diluent was back-filled to a final concentration of 0.3% (v/v). Cell plates were incubated for 48 h at 37°C (95% humidity, 5% CO_2_). 50 μL cell suspension from each well was transferred into the wells of a white polystyrene plate (Corning). Cell viability was assessed with CellTiter-Glo 2.0 reagent (Promega), as above. Data were imported into and analyzed using GraphPad Prism software. The luminescence values were scaled to the average of the RSL3-DMSO-treated group (defined as 0%) and the average of the DMSO-treated group (defined as 100%). EC_50_ values for each compound were estimated using standard four-parameter curve fitting algorithms.

### Iron-BSO cell survival assays

Ferroptotic cell death was induced in patient-derived primary fibroblast cells using L-buthionine-(*S*,*R*)-sulfoximine (BSO, Sigma) in combination with iron(III) citrate (FeC, Sigma). For each patient-derived fibroblast culture evaluated, an initial matrix of BSO and FeC titrations was performed, from which the minimal BSO and FeC concentrations were determined that (a) resulted in >80% reduction in cell viability and (b) showed the optimal differential sensitivity compared to a reference pediatric healthy fibroblast control (GM00038, tested in parallel with each experimental replicate). Under these optimal patient fibroblast-specific challenge conditions, subsequent EPI-743 rescue assays were performed, as follows.

Fibroblasts were seeded at 2,500 cells per well in 96 well clear bottom tissue culture-treated polystyrene plates (Falcon) using an electronic multichannel pipette. Cells were incubated for 5 h at 37°C (95% humidity, 5% CO_2_) to allow attachment. FeC and EPI-743 were administered to the desired final concentrations using an electronic multichannel pipette, with DMSO final concentration maintained at 1% (v/v). Cell plates were incubated for 18 h at 37°C (95% humidity, 5% CO_2_). BSO was then added at the desired final concentration and cell plates were incubated for 48 h at 37°C (95% humidity, 5% CO_2_). After removing media, cells were washed once with Phosphate Buffered Saline with Magnesium and Calcium (PBS++, Gibco). PBS++ containing 1 μM Calcein AM (Anaspec) was then dispensed to the wells. The cell plates were incubated at 37°C (95% humidity, 5% CO_2_) for 30 min. The relative fluorescence intensity (RFU) was measured using a SpectraMax M2 plate reader (Excitation/Emission = 488 nm/525 nm). Calcein fluorescence values were standardized to the average of the FeC-BSO/DMSO group (defined as 0% viability) and the average of the DMSO control group (defined as 100% viability). EC_50_ values were calculated using standard four-parameter curve fitting algorithms in GraphPad Prism or XLfit.

Ferroptotic cell death was induced in *CDKL5* B-lymphocytes using BSO and FeC. To assess compound rescue activity and potency from ferroptotic cell death, cells were seeded at 40,000 cells per well in 96 well clear bottom tissue culture-treated polystyrene plates (Corning) using an electronic multichannel pipette. EPI-743 was administered to the desired final concentrations using a D300e Digital Dispenser (Tecan). DMSO diluent was back-filled to a final concentration of 0.3% (v/v). BSO and FeC were then added to the wells at the desired final concentration. Cell plates were incubated for 48 h at 37°C (95% humidity, 5% CO_2_). 50 μL cell suspension from each well was collected into the wells of a white polystyrene plate. Viability was assessed using CellTiter-Glo 2.0 reagent (Promega), as above. The luminescence values were standardized to the average of the FeC-BSO/DMSO group (defined as 0% viability) and the average of the DMSO group (defined as 100% viability). Data was imported into and analyzed using GraphPad Prism software. EC_50_ values for each compound were estimated using standard four-parameter curve fitting algorithms.

### RSL3 cell lipid oxidation assay

Ferroptosis was induced by RSL3 (2 μM) in patient-derived fibroblast cells as described above, except seeding at 2,800 cells per well in clear bottom, black wall, 96 well, tissue culture-treated polystyrene microplates. The rate of cellular lipid oxidation after GPX4 inhibition was assessed by monitoring the time-dependent changes in green fluorescence of cells pre-labeled with 5 μM BODIPY 581/591 C11 dye (ThermoFisher) for 30 min. Images were acquired with the IncuCyte S3 live-cell imaging apparatus (Sartorius) using a 10x objective, and 440–480 nm Excitation / 504–544 nm Emission filters. Cells were imaged once hourly for up to 24 h after treatment. A minimum of two images were acquired per field, and at least three replicate wells were imaged per condition in each experiment. Quantification of cell images was performed using the IncuCyte Zoom software using the Basic Analyzer tool. Total green positive area per well was defined relative to the local background-subtracted fluorescent signal. For the lipid oxidation rate, the initial rate of change in thresholded fluorescence was calculated (0–5 h), then expressed relative to that observed in the RSL3-only control treatment group, which was defined as 100%. For cell lipid oxidation time course studies, the background-subtracted fluorescent signal was normalized to the initial time point, then plotted longitudinally.

### *In vitro* cell quinone reduction measurement

PCH6 patient fibroblasts were seeded at 150,000 cells per well in 6 well tissue-culture treated plates in standard culture medium for 18 h prior to compound addition. After the specified treatment time, the culture medium was removed and adherent cells were washed with PBS. The wash was removed and adherent cells were harvested by adding 1 mL of 100 mg/mL succinic anhydride in 95:5 (acetonitrile:triethylamine), followed by immediate cell scraping. Cell samples were then transferred to a 1.5 mL Eppendorf lo-bind tube, vortexed for 15 sec, and kept on wet ice until all samples were harvested. Samples were vortexed again for 15 sec and centrifuged in an Eppendorf 5427 centrifuge at 13,500 X g, 4°C, for 3 min.

Following centrifugation, samples were protein-precipitated by addition of 3 volumes of acetonitrile. Following precipitation, samples were vortexed for 30 seconds and centrifuged in a Sorvall Legend XFR centrifuge at 4,000 X g, 4°C, for 10 min. Subsequently, 1 volume of supernatant was transferred to a 384-well injection plate and diluted with 1 volume of mobile phase A and pipette mixed. Internal standard Diclofenac was added to the 384-well injection plate. The injection plate was vortexed and centrifuged for a final time at 4,000 X g, 4°C, for 10 minutes and subsequently placed in a Shimadzu Nexera MP autosampler maintained at 4°C for the duration of the analytical run.

Analytes were chromatographically separated on a Phenomenex Kinetex C_18_ (2.1 x 50 mm, 1.3 μm) column using a reverse phase gradient. The composition of mobile phase A was 0.1% formic acid in water. The composition of mobile phase B was 3:1 (0.1% formic acid in (acetonitrile: isopropyl alcohol)). Mobile Phase B was ramped from 30% to 98% over 7 min. The total run time was 8 min.

Sample concentrations were analyzed using a developed method on a Shimadzu Nexera MP system coupled to an AB Sciex 6500+ Qtrap mass spectrometer and configured for micro-flow. The MS/MS instrument was operated in positive ESI mode with a source temperature of 250°C. Electrospray conditions for the μLC-ESI-MS/MS method were optimized and were as follows: Ionspray voltage was set to 5500 V, temperature of 250°C, curtain gas of 20, CAD gas of 10, and ion source gas 1 and 2 of 15 and 60 psi respectively. Analyzer parameters were optimized for each compound using a combination of manual tuning and compound optimization. The mrm transitions were 423.251→165.100 for EPI-743, 425.230→165.100 for EPI-743 hydroquinone, 625.154→265.00 for EPI-743 hydroquinone bis-succinate, and 295.942→214.000 for IS-Diclofenac. Uncapped EPI-743 hydroquinone was monitored but not observed in samples.

Data were analyzed using Sciex Analyst Chromatography software, version 1.6.3 and Sciex MultiQuant software. The standard curve equation (y = mx +b) is generated from the calibration standards with weighted regression.

### Targeted LC-MS/MS analysis of cellular HETEs

Fibroblasts were cultured in 6 well (120,000 or 240,000 cells per well) or 24 well (24,000 cells per well) plates, followed by RSL3 treatment, as above, to initiate ferroptosis. At the time of harvest, 6 mL of methanol (-80°C) was added directly to each well of the cell plate. The plate was gently shaken before the media:methanol (1:3, v:v) solution was aspirated. The cell plate was then frozen on dry ice and transferred to -80°C storage.

Cell plates were removed from -80°C storage and placed in a dry ice bucket. To each well of the cell plate was added 800 μL of 2:1 methanol:75 mM HCl containing 50 nM of isotopically labeled internal standards. The cells were mechanically scraped with a Costar cell lifter and then transferred to a 5 mL 48-well plate. Lipids were extracted with two serial additions of 1.6 mL Heptane:Isopropyl Acetate (4:1, v:v) containing 0.4 mM butylated hydroxytoluene. Following centrifugation for 5 minutes at 2,000 X g (4°C), the organic layer was transferred to a glass-lined plate. The extracted lipids were evaporated to dryness under nitrogen without heating. All samples were reconstituted in methanol and transferred to injection plates for mass spectrometry analysis.

A Shimadzu Nexera MP system coupled to a Sciex 6500 Qtrap and configured for micro-flow was used to analyze all samples. Samples were maintained at 4°C in the Nexera MP autosampler for the duration of the analytical run.

A Thermo Gold-C_8_ column (1.9 μm, 1.0 x 100 mm) operated at 45°C was used for chromatographic separation. The total run time of the liquid chromatography method was 10 min. Mobile phase A consisted of water with 0.1% formic acid. Mobile phase B was composed of 0.1% formic acid in 3:1 acetonitrile:isopropyl alcohol. Gradient elution was employed, with mobile phase B being ramped from 5% at 0 min, to 50% at 2 min, and finally 98% at 7.5 min. Electrospray conditions for the μLC-ESI-MS/MS method were optimized for a flow rate of 125 μL/min and were as follows: Ionspray voltage was set to -4500 V, temperature of 250°C, curtain gas of 20, CAD gas of 10, and ion source gas 1 and 2 of 20 and 40 psi, respectively. Analyzer parameters were optimized for each compound using a combination of manual tuning and compound optimization.

### siRNA knockdown of *ALOX15*

Pools of siRNA against human *ALOX15* (L-003808-00) and non-targeting control (D-001810-10; referred to as siControl) were purchased from Dharmacon. Using standard protocols, Lipofectamine RNAiMAX transfection reagent in Opti-MEM media (Thermo/Invitrogen) was used to transfect cells with 0.01 pmol siRNA per well of a 6-well plate. Media was exchanged 24 h after transfection to reduce the likelihood of negative impact of RNAiMAX transfection reagent on cellular physiology. 72 h after transfection, cells were used for BODIPY 581/591 C11 lipid oxidation assays and RSL3 survival assays as described above. Data was imported into and analyzed using GraphPad Prism 8.0.2 software. The luminescence values were scaled to the DMSO-treated wells and the 10 μM RSL3-treated wells within a given experiment (defined as 100% and 0%, respectively). RSL3 IC_50_ values for each siRNA knockdown were estimated using standard four-parameter curve fitting algorithms. Area under the curve (AUC) and Standard Error (SE) values were calculated for the scaled 42-point dose-response values from three independent experimental repeats, then compared by unpaired t-test relative to siControl.

### Quantitative RT-PCR measurement of *ALOX15* mRNA levels

Total RNA from roughly 300,000 cells was extracted using the NucleoSpin RNA Plus kit (Macherey-Nagel). cDNA was prepared using 1 μg of total RNA with the Superscript IV VILO kit at 60°C (ThermoFisher). cDNA was diluted 5-fold and used in downstream qPCR reactions with PerfeCTa Fast Mix II (Quanta) and TaqMan probes (ThermoFisher) on a LightCycler480 instrument (Roche). *PPIA* (Hs99999904_m1) was used as the endogenous control gene to determine knock down efficiency for *ALOX15* (Hs00993765_g1). Reactions were run in technical triplicate. Data was analyzed in Excel using the ΔΔCt method.

### Enzymology materials

Purified human 15-lipoxygenase-1 (15-LO) was expressed and purified by Proteos, Inc., similar to previously published protocols [45]. Briefly, 6xHis-tagged human 15-LO was expressed in SF9 cells via baculovirus expression and purified by nickel affinity chromatography followed by size exclusion chromatography. The assay buffer used was DPBS (Gibco). Sodium cholate and sulfuric acid were obtained from Sigma-Aldrich. Arachidonic acid was obtained from Cayman Chemical. Xylenol orange disodium salt and ammonium iron (II) sulfate hexahydrate were obtained from Fluka. HPLC-grade methanol was obtained from Millipore Sigma.

### Lipoxygenase enzyme inhibition assay

The activity of 15-LO was determined by using a modified version of the ferrous oxidation-xylenol orange (FOX) assay [46]. FOX reagent was prepared by combining 1 part reagent A (10 mM ammonium iron (II) sulfate in 2.5 M sulfuric acid) with 99 parts reagent B (430 μM xylenol orange in 90% (v:v) methanol, 10% water). To obtain fully active 15-LO, 3 μM enzyme was pre-incubated with 10 μM ammonium iron (II) sulfate in 1x DPBS on ice for 30 minutes. Enzyme reactions (500 μL final volume) were conducted in 1.0 mL deep well plates in 1x DPBS, contained final concentrations of 50 μM arachidonic acid as a substrate, 0.05% (w:v) sodium cholate, 10% (v:v) DMSO, and were initiated by the addition of 1 μM 15-LO. Stock solutions of EPI-743 quinone or hydroquinone were prepared in DMSO at 10x concentrations and 50 μL was added to enzyme reaction mixes to obtain a final concentration of 10% DMSO. After initiation, 50 μL of the reaction was removed every 30 seconds over the course of 4 minutes, quenched by addition to 50 μL FOX reagent in Greiner clear 96–well half area plates, and incubated for 30 minutes. Linear rates were assessed via FOX assay absorbance change at 560 nm on a Molecular Devices Spectramax M2.

### Statistical methods

Unless otherwise specified, statistical analyses were performed using GraphPad Prism for Windows (versions 7.0.2, 7.0.3, or 8.0.2). Standard 4-parameter curve fitting was used to calculate EC_50_ and IC_50_ values, along with 95% confidence bands when specified. Areas under the dose-response curves in siRNA experiments were calculated on log-transformed concentrations. To determine if groups showed normal distributions, a D’Agostino & Pearson normality test was used. If any group within a given figure did not meet normality assumptions, then a non-parametric Kruskal-Wallis test with Dunn’s multiple comparisons test was used. When only two groups were compared, a two-tailed, unpaired t-test was used. The threshold for statistical significance was defined as *p*<0.05. *p-*values were displayed in figures as ***, *p*<0.001, and ns, *p*>0.05.

## Results

### GPX4 inhibition by RSL3 induces ferroptosis in PCH6 patient-derived skin fibroblasts

We initially established ferroptosis assays in skin fibroblasts derived from three different PCH6 patients. All three patients’ cells had sequence changes predicted to introduce frame shift, nonsense, and/or deleterious missense mutations in the *RARS2* gene (Table 1).

**Table 1:** PCH6 patient-derived fibroblasts studied.

*Subject*	*Patient Sex*	*Patient Age at Biopsy (y)*	*RARS2 Genotype*
1			c.943C>T, p.R315Ter c.601C>G, p.H201D *in trans*
	Female	1.33	
	
2			c.419T>G, p.F140C c.1612delA, p.T538fs *in trans*
	Female	13.7	
	
3			c.419T>G, p.F140C c.1612delA, p.T538fs *in trans*
	Female	11.9	
	

**Fig 1:**
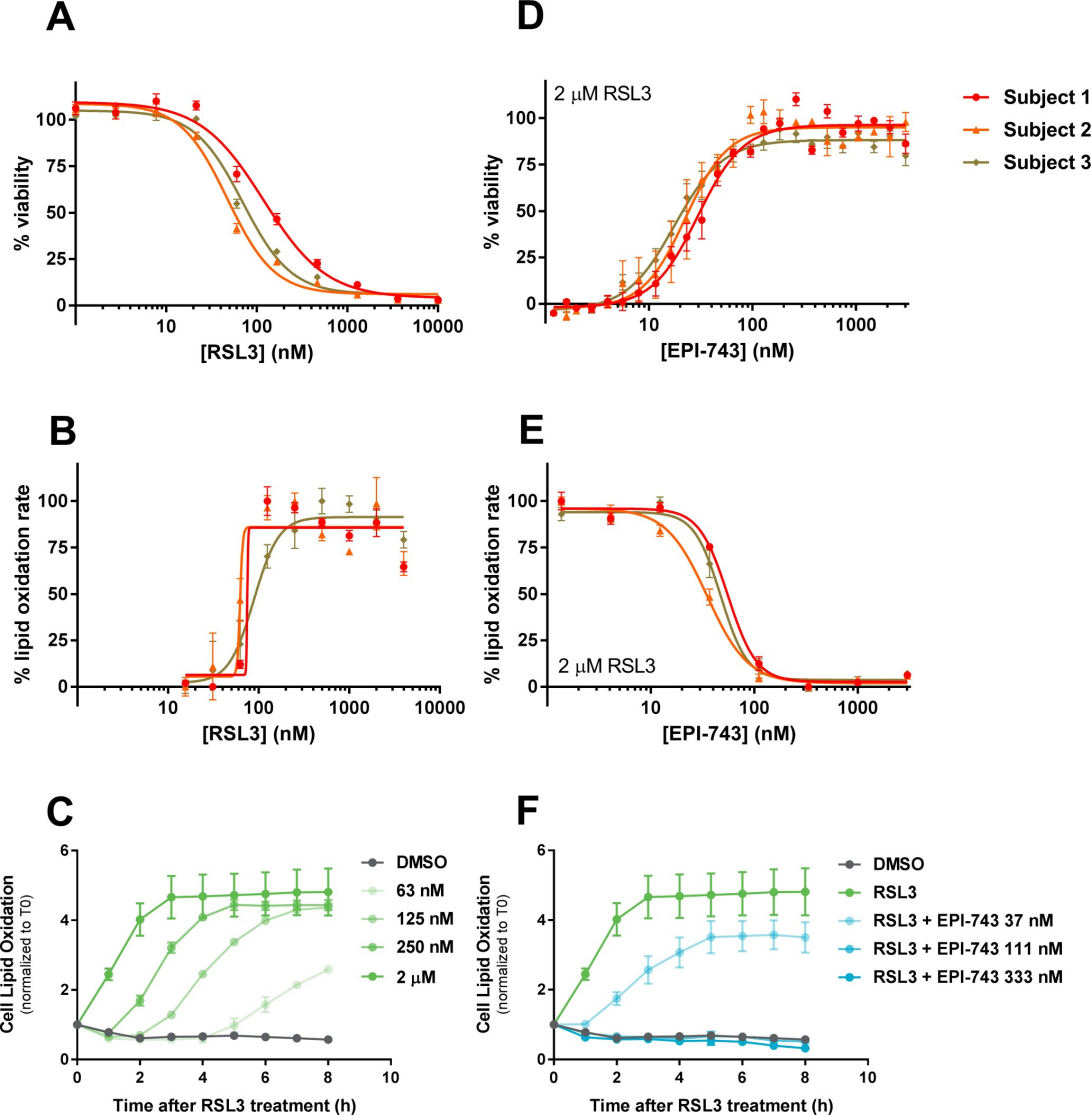
EPI-743 rescues PCH6 patient fibroblasts from RSL3-induced lipid oxidation and ferroptosis. (A) Dose-dependent reduction in viability of PCH6 patient cells by RSL3 treatment (N = 6–8 replicates for each patient cell summarized). Cell viability was assessed 18 h after RSL3 treatment using CellTiter-Glo 2.0 reagent to quantify cellular ATP. (B) The potency of RSL3-induced BODIPY 581/591 C11 lipid oxidation mirrors that of cytotoxicity (N = 6 replicates per patient culture). (C) Dose- and time-dependent BODIPY 581/591 C11 lipid oxidation in PCH6 patient fibroblasts, as measured by time-lapse microscopy (N = 2 independent experiments per patient culture, representative data from Subject 1 shown). (D) EPI-743 dose-dependently protected PCH6 patient fibroblasts from ferroptosis induced by RSL3 (2 μM, 18 h). Dose-response curves are representative of 5–9 independent experiments summarized in Table 2. (E) EPI-743 dose-dependently decreased the rate of cellular BODIPY 581/591 C11 oxidation following RSL3 (2 μM) treatment (N = 6 replicates per patient culture). (F) Time course of EPI-743 prevention of BODIPY 581/591 C11 lipid oxidation (N = 2 independent experiments per patient culture, representative data from Subject 1 shown). Mean ± SEM displayed along with best 4-parameter curve fits (A, B, D, E).

To induce ferroptosis, each PCH6 patient fibroblast culture was treated with the irreversible GPX4 inhibitor RSL3 [44] and cellular viability was assessed 18 h later. RSL3 caused dose-dependent decreases in cell viability, with IC_50_ values ranging from 53±4 to 139±48 nM (mean±SD, N = 3–4 independent experiments for each patient culture; Fig 1A). While the potency of RSL3 was comparable in the PCH6 patient-derived fibroblasts and a panel of seven reference pediatric apparently healthy control fibroblast cultures, the maximal RSL3-induced toxicity was nominally enhanced in PCH6 patient fibroblasts compared to those from healthy controls (95±1% (N = 3) vs. 71±17% (N = 7) reduction in cell viability by 2 μM RSL3, respectively; mean±SD, p<0.01, Mann-Whitney test; S1 Fig).

**Table 2:** Summary of EPI-743 rescue potencies in PCH6 patient fibroblasts.

	EPI-743 potency (nM)		
	*RSL3*	*RSL3*	*FeC / BSO*
	*Challenge*	*Challenge*	*Challenge*
Subject	Survival EC_50_	Lipid Oxidation IC_50_	Survival EC_50_
	*mean ± SD [N]*	*mean ± SD [N]*	*mean ± SD [N]*
1	21.8 ± 10.2	57.6 ± 11.3	9.6 ± 5.6
	[9]	[3]	[2]
2	17.8 ± 5.4	33.1 ± 3.3	4.8 ± 4.9
	[8]	[3]	[2]
3	17.3 ± 6.9	41.0 ± 3.0	4.8 ± 3.3
	[5]	[3]	[2]

After establishing dose-dependent sensitivity to RSL3-mediated ferroptosis, subsequent experiments focused on the mechanisms of ferroptotic cell death and rescue in PCH6 patient-derived fibroblasts. Lipid oxidation is a hallmark of ferroptotic cell death [9] that can be assessed using the oxidation-sensitive fluorescent lipid probe BODIPY 581/591 C11. RSL3 treatment of PCH6 patient fibroblasts dose-dependently increased cellular lipid oxidation within ~1 hour of compound exposure, as measured by time-lapse fluorescence microscopy (Fig 1B and 1C). The potency of RSL3-induced BODIPY 581/591 C11 lipid oxidation was similar to that observed for the loss of cell viability (lipid oxidation EC_50_ range 103±29 to 134±36 nM, mean±SD, N = 3 independent experiments per patient culture; Fig 1B).

### EPI-743 prevents RSL3-induced ferroptosis in PCH6 patient fibroblasts

The activity and potency of EPI-743 was evaluated in PCH6 patient-derived fibroblast ferroptosis assays. EPI-743 co-treatment completely prevented the ferroptotic cell death induced by RSL3 (2 μM) in all three Subjects’ fibroblasts, with mean EC_50_ potency values of 17.3 to 21.8 nM (Fig 1C and 1D and Table 2). EPI-743 also dose-dependently prevented RSL3-induced oxidation of cellular BODIPY 581/591 C11, with mean IC_50_ values of 33.1 to 57.6 nM (Fig 1E and 1F and Table 2). EPI-743 therefore protects PCH6 patient-derived fibroblasts from lipid oxidation and ferroptosis induced by irreversible pharmacological inhibition of GPX4.

### Ferroptosis induced by glutathione depletion and iron overload in PCH6 patient fibroblasts is also prevented by EPI-743

**Fig 2:**
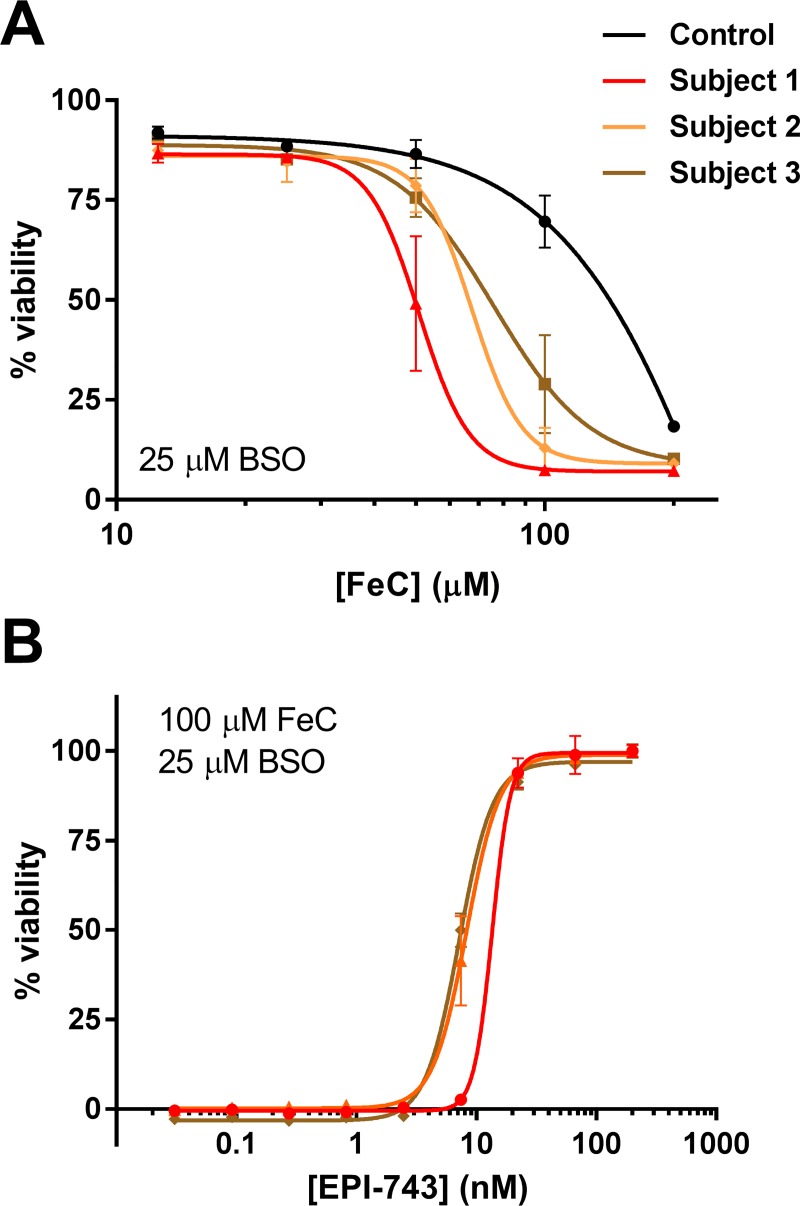
EPI-743 rescues PCH6 patient fibroblasts from iron / BSO-induced ferroptosis. (A) PCH6 patient fibroblasts were sensitive to ferroptosis induced by BSO-mediated GSH depletion in the presence of varying concentrations of added iron(III) citrate (FeC). Viability relative to untreated control wells was measured by Calcein AM assay 48 h after BSO treatment. (B) EPI-743 treatment dose-dependently prevented FeC/BSO-induced ferroptosis in PCH6 patient fibroblasts. Mean ± SEM (N = 3) along with best-fit 4-parameter curve fits displayed; representative of two independent experiments. Refer to Table 2 for summary statistics of calculated rescue potencies.

To complement the model of acute RSL3-mediated pharmacological inhibition of GPX4 and provide further evidence of ferroptosis-related cellular pathology, we employed a second method of inducing ferroptosis that models the GSH depletion and oxidative stress observed in mitochondrial disease patients [47]. Specifically, we utilized the inhibitor of γ-glutamylcysteine ligase, buthionine sulfoximine (BSO), to deplete cellular GSH in the presence of excess free iron (iron(III) citrate, FeC). Treatment of PCH6 patient fibroblasts with FeC and BSO resulted in a gradual ferroptotic cell death by 48 h post-treatment. All three PCH6 patient fibroblasts were more sensitive to the FeC / BSO challenge compared to a reference pediatric control fibroblast culture (GM00038, Coriell; Fig 2A). As with RSL3-induced ferroptosis, EPI-743 potently and completely rescued PCH6 patient cells from FeC / BSO-induced ferroptosis (rescue EC_50_ range 4.8 to 9.6 nM; Fig 2B and Table 2). Thus, EPI-743 protected PCH6 patient fibroblasts from ferroptosis, whether induced by acute GPX4 inhibition or by persistent GSH depletion in the presence of FeC.

### EPI-743 is reduced to its hydroquinone form in PCH6 cells and inhibits human 15-lipoxygenase (15-LO)

**Fig 3:**
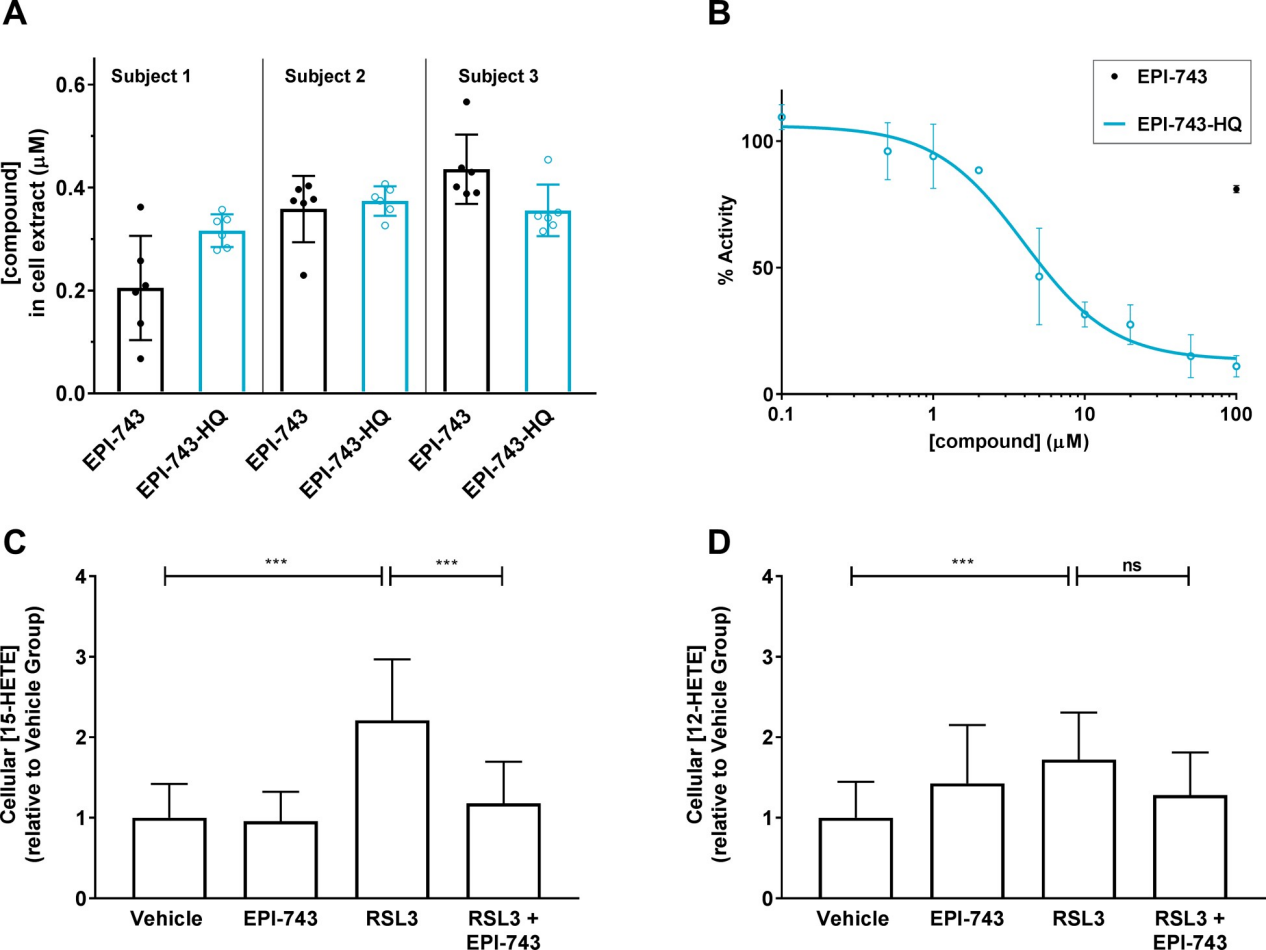
EPI-743 hydroquinone is detected in PCH6 patient fibroblasts and inhibits human 15-LO. (A) Dosed EPI-743 (1 μM, 3 h) was reduced to EPI-743 hydroquinone (EPI-743-HQ) in PCH6 patient fibroblasts from Subjects 1, 2, and 3. Mean ± SD displayed, N = 6 per subject. (B) The activity of purified human 15-LO was monitored via a ferrous oxidation-xylenol orange (FOX) method. EPI-743-HQ dose-dependently inhibited human 15-LO enzyme activity, whereas EPI-743 showed only 20% inhibition at 100 μM. Mean ± SD displayed, N = 2. The IC_50_ for the displayed curve is 4.4 μM. (C, D) LC-MS/MS measurement of downstream products of 15-LO (15-HETE, C) and 12-LO (12-HETE, D) in PCH6 patient (Subject 1) fibroblasts following RSL3 (2 μM, 4 h) challenge, without or with EPI-743 (200 nM) co-treatment. Compound was dosed in the quinone form. Results shown are N = 17 replicates from three independent experiments. Mean ± SD displayed. ***, p<0.001; ns, not significant; by Kruskal-Wallis test followed by Dunn’s multiple comparisons test. We noted that cellular 15-hydroperoxyeicosatetraenoic acid (15-HpETE), the direct product of 15-LO acting upon arachidonic acid as substrate, was not consistently detected within the quantifiable range by LC-MS/MS in our studies.

We previously reported [17] that the reduced hydroquinone metabolite of EPI-743 (referred therein as α-tocotrienol hydroquinone or αT3HQ) directly inactivates a 15-LO enzyme via reduction of its non-heme iron from its active Fe^3+^ state to an inactive Fe^2+^ state, providing one plausible mechanism for the prevention of cellular lipid oxidation and ferroptosis in PCH6 cells. To test this hypothesis, we first confirmed that EPI-743 is indeed metabolized in PCH6 patient fibroblasts to its reduced hydroquinone form (EPI-743-HQ; Fig 3A), with an average of 45–62% of dosed compound detected as EPI-743-HQ (n = 6/patient culture). Second, we demonstrated that the EPI-743-HQ metabolite dose-dependently inhibited purified human 15-LO enzyme activity, while the parent EPI-743 had only weak (20%) inhibitory activity at 100 μM (Fig 3B). Third, we demonstrated that RSL3 treatment of PCH6 patient fibroblasts significantly elevated cellular levels of the 15-LO downstream product 15-hydroxyeicosatetraenoic acid (15-HETE) that were completely prevented by EPI-743 co-treatment (200 nM; Fig 3C). By contrast, the significant RSL3-dependent increase in cellular levels of the 12-lipoxygenase downstream product, 12-hydroxyeicosatetraenoic acid (12-HETE), was not prevented by EPI-743 co-treatment (Fig 3D). Together, these data support our model that the ferroptosis protection conferred by EPI-743 in PCH6 patient-derived fibroblasts results from the selective inhibition of 15-LO.

### *ALOX15* knockdown in PCH6 patient fibroblasts prevents RSL3-mediated lipid oxidation and ferroptosis

**Fig 4:**
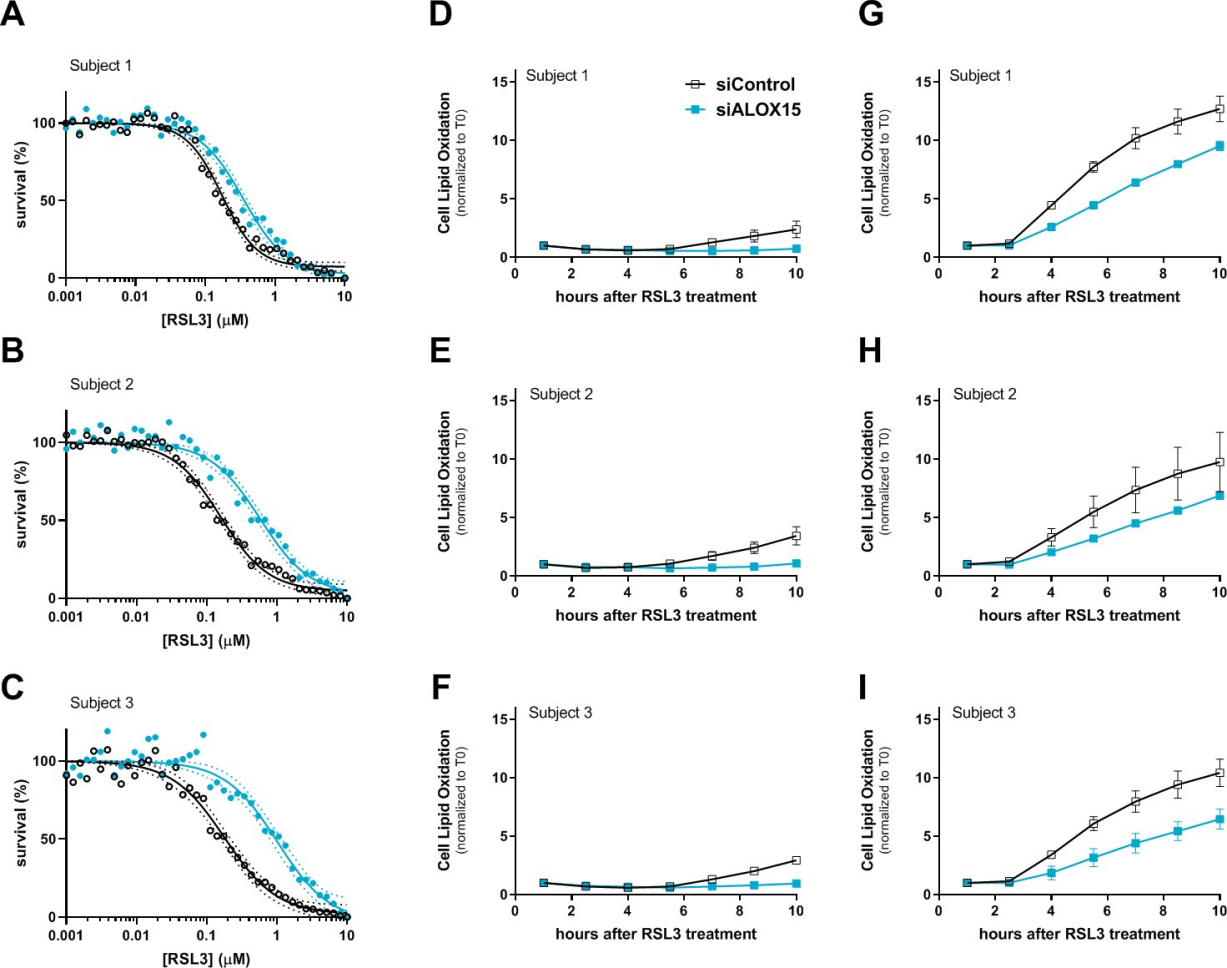
*ALOX15* siRNA-mediated gene knockdown partially abrogates ferroptosis in PCH6 patient fibroblasts. (A-C) siRNA knockdown of *ALOX15* (siALOX15) decreased the sensitivity of PCH6 patient cells to RSL3-induced cytotoxicity, as assessed by ATP levels at 18h. Summarized results in each PCH6 patient culture from three independent knockdown experiments. Means (circles) and best-fit curves (solid lines) from 4-parameter fits with associated 95% confidence bands (dotted lines) displayed. (D-I) siRNA-mediated knockdown of *ALOX15* partially prevented BODIPY 581/591 C11 lipid oxidation induced by RSL3 (60 nM, panels D-F, or 167 nM, panels G-I) in fibroblasts from Subject 1 (D, G), Subject 2 (E, H), or Subject 3 (F, I). Cellular lipid oxidation was measured by changes in green fluorescence by time-lapse video microscopy. Mean ± SEM displayed, N = 6–8 per group. Representative results from two independent studies. Statistical comparison of Area Under the Curve values was performed by unpaired t-test relative to siControl; see S1 Table for summary of results. *ALOX15*-targeted siRNA transfection resulted in >90% reduction in the target mRNA, as assessed by quantitative RT-PCR.

In rodent cell-based studies, other investigators have demonstrated that inactivation of specific lipoxygenases can slow or prevent cell death induced by GPX4 inactivation or GSH depletion, although the responsible lipoxygenase(s) appears to depend on cellular context [9,11]. To further substantiate a specific role of 15-LO in RSL3-mediated ferroptosis in PCH6 patient-derived fibroblasts, we employed siRNA-mediated knockdown of *ALOX15*. In each patient culture, treatment with *ALOX15*-directed siRNA decreased the cells’ sensitivity to an RSL3 insult (Fig 4A–4C, refer to S1 Table for full statistical analysis of results). Furthermore, siRNA-mediated inhibition of *ALOX15* partially mitigated RSL3-induced cellular lipid oxidation (Fig 4D–4I). These findings provide convergent support for the model that EPI-743 can protect PCH6 patient-derived fibroblasts from RSL3-induced ferroptosis via inhibition of 15-LO activity via its hydroquinone metabolite.

### EPI-743 potently prevents ferroptosis in primary cells derived from additional pediatric diseases associated with epilepsy

**Fig 5:**
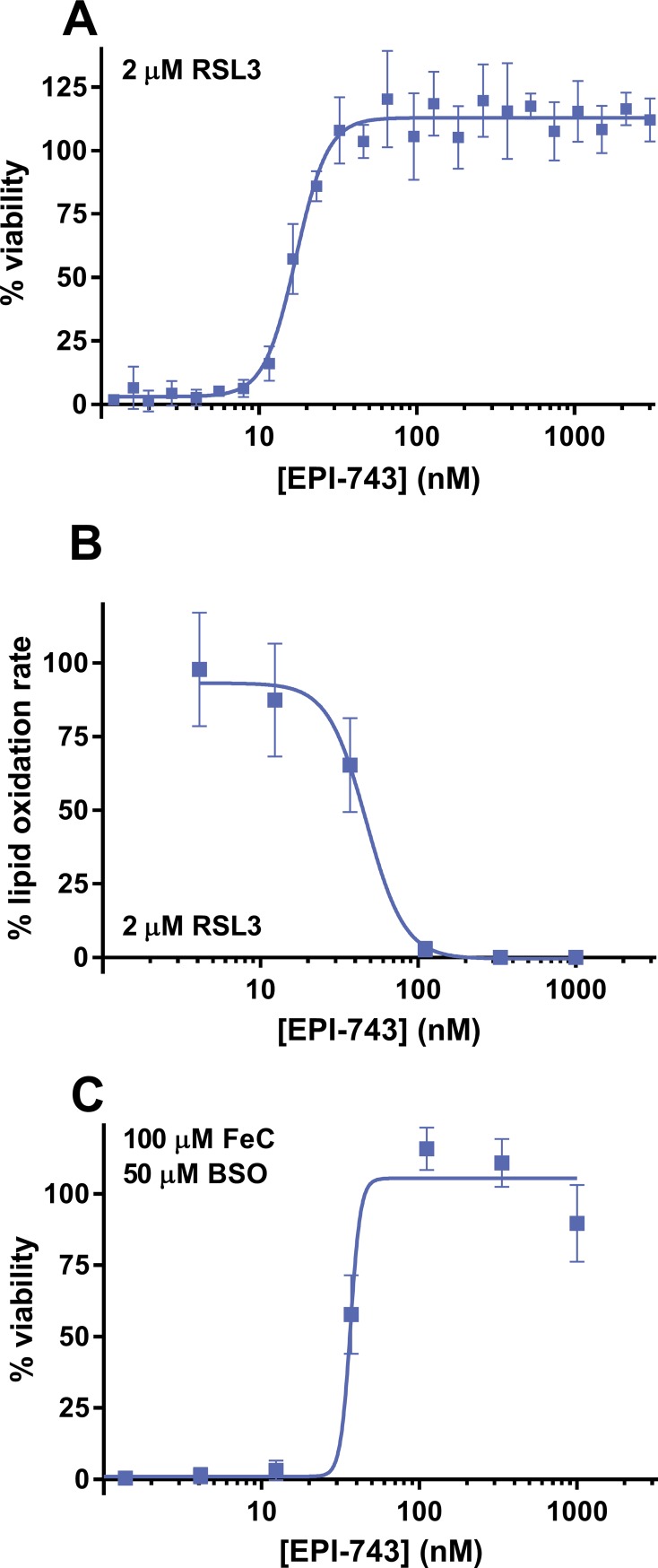
EPI-743 prevents ferroptosis and lipid oxidation in Leigh syndrome patient-derived fibroblasts. (A) Dose-dependent protection by EPI-743 from RSL3-induced cytotoxicity of Leigh syndrome patient-derived fibroblasts (Subject 4) as assessed by CellTiter-Glo 2.0. (B) Dose-dependent protection by EPI-743 from RSL3-induced lipid oxidization as assessed by BODIPY 581/591 C11 cellular fluorescence. (C) Dose-dependent protection by EPI-743 from FeC/BSO-induced cytotoxicity as assessed by Calcein AM fluorescence. Mean ± SD displayed, N = 6.

We extended our evaluation of the anti-ferroptosis activity of EPI-743 to fibroblasts derived from patients with additional pediatric diseases characterized by high epilepsy incidence, such as Leigh syndrome (OMIM #256000), Alpers syndrome (OMIM #203700), and Rett syndrome (OMIM #312750). Each patient cell culture was assayed under FeC/BSO challenge conditions under which the patient cells demonstrated increased sensitivity relative to reference healthy control culture(s) (S1 and S2 Figs). As summarized in Table 3, EPI-743 potently protected fibroblasts derived from Leigh syndrome (see Fig 5C for representative dose-response rescue curve for Subject 4), Alpers syndrome, and Rett syndrome patients from ferroptosis induced by GSH depletion and excess iron. EPI-743 treatment also protected additional patient-derived fibroblasts from RSL3-induced lipid oxidation and ferroptosis (Table 3, S3 Fig and Fig 5A and 5B). Ferroptosis protection was not limited to patient fibroblasts: EPI-743 also protected transformed B-lymphocytes derived from a patient with early infantile epileptic encephalopathy type 2 (EIEE2, OMIM #300672) and mitochondrial Complex IV deficits due to a partial deletion of the *CDKL5* gene (Table 3 and S3 Fig). In total, these patient-derived cell culture results clarify a cellular mechanism-of-action for EPI-743 and support ongoing clinical investigations of EPI-743 in additional pediatric epilepsy disorders.

**Table 3:** Summary of EPI-743 ferroptosis rescue of cells derived from pediatric seizure disorder patients.

				EPI-743 Potency (nM)		
				*RSL3* *Survival*	*RSL3* *Lipid Oxidation*	*FeC/BSO* *Survival*
Condition	Gene	Cell ID	Cell type	*EC*_*50*_	*IC*_*50*_	*EC*_*50*_
	*MT-ND3*	4	F	18	47	37
Leigh syndrome	Unknown	GM03672	F	28	20	22
	Unknown	GM01503	F	20	19	35
Alpers	*POLG*	5	F	-	-	19
syndrome	*POLG*	070718	F	-	-	14
Rett syndrome	*MECP2*	GM17567	F	46	32	34
Epileptic Encephalopathy early infantile, 2 (EIEE2)	*CDKL5*	GM23710	B	90	-	20

F, dermal fibroblast

B, immortalized B lymphocytes

-, not tested

## Discussion

### Loss of ferroptosis regulation leads to seizure phenotypes: Evidence from mouse genetic studies

Evidence for ferroptosis as an underlying facet of epilepsy is emerging from mouse genetic models with seizure phenotypes, particularly those in which the function of the essential phospholipid hydroperoxide-reducing GPX4 enzyme was targeted directly or indirectly. Neuronal-specific *Gpx4* loss-of-function results in touch-evoked seizures, cerebellar hypoplasia, astrogliosis, and loss of parvalbumin-positive (PV+) interneurons [9,48,49]. The *Gpx4*^*cys/cys*^ model, in which the catalytic selenocysteine of GPX4 is replaced with cysteine, elegantly confirms an essential role for *Gpx4* catalytic activity in the etiology of seizure and neuroinflammation: *Gpx4*^*cys/cys*^ mice also have spontaneous perinatal seizures, PV+ interneuron loss, reactive astrogliosis, and microgliosis [12]. Intriguingly, a similar constellation of phenotypes was also described in the neuronal tRNA[Ser]^Sec^ *Trsp* mutant mouse, which has reduced GPX4 activity: *Trsp* mutant mice also develop seizures and exhibit PV+ interneuron loss, astrogliosis, and cerebellar hypoplasia, including a marked loss of Purkinje cells [48,49]. The PV+ GABAergic interneurons most impacted by *Gpx4* and *Trsp* deficiency contain relatively high numbers of mitochondria and ETC proteins to support their elevated rates of firing activity [1,50,51], which makes this class of interneurons exceptionally vulnerable to mitochondrial oxidative stress [52,53]. PV+ interneurons serve as key regulators of cortical and hippocampal network activity and excitatory output, suggesting a possible mechanism for seizure origination in *Gpx4*- and *Trsp*-deficient brains. In summary, *Gpx4* and *Trsp* mouse models suggest a link between seizures and underlying genetic deficits in the biochemical mechanisms used to clear ferroptosis-promoting oxidized lipids.

### Loss of ferroptosis regulation leads to epilepsy syndromes: Evidence from human studies

Genetic evidence linking ferroptosis, neuroinflammation, and epilepsy also arises from human genetic disorders involving selenoenzymes. Sedaghatian-type spondylometaphyseal dysplasia (OMIM #250220) is an ultrarare pediatric disorder caused by mutations in the *GPX4* gene characterized by seizures and cerebellar hypoplasia [20–22]. Wirth and colleagues also noted striking parallels between their *Trsp* mutant mice and human patients with pontocerebellar hypoplasia type 2D (PCH2D, OMIM #613811), a syndrome caused by deleterious variants in the *SEPSECS* gene, which encodes a protein essential to selenoprotein biosynthesis [49,54,55]. PCH2D patients present with infantile spasms and progressive cerebellar atrophy accompanied by Purkinje cell loss and astrogliosis [56]. Reports of dietary selenium deficiency leading to intractable seizures provide additional evidence for a direct mechanistic role of selenium-regulated ferroptosis in epilepsy [57,58].

Intriguingly, the clinical features of PCH2D have been noted to overlap with those of PCH6 and other disorders arising from genetic defects in mitochondrial translation components [59]. Indeed, Purkinje cell loss and astrogliosis were reported in PCH6 and Alpers syndrome patient brains studied postmortem [23–25]. Collectively, these results are consistent with ferroptosis being an underlying (and perhaps underappreciated) aspect of seizure disorders, including those associated with mitochondrial defects, and further suggest that mitigating ferroptosis may be a promising therapeutic strategy for the treatment of epilepsy.

### Mitochondrial dysfunction is commonly associated with epilepsy

Once thought to be primarily a disease of ion channels and neurotransmitter-mediated signal transduction, emerging clinical evidence shows that epilepsy can arise due to mutations in mtDNA or nuclear-encoded components of the mitochondria [1]. Primary defects in mtDNA maintenance, mitochondrial protein translation, ETC assembly, or respiratory chain function can each result in inefficient ATP production and oxidative stress, manifesting in some patients as clinical presentation of high seizure incidence [4]. Neurons appear to be particularly susceptible to mitochondrial defects, likely due to the stringent ATP demands for depolarization and synaptic transmission. We speculate that as mitochondrial function drops beneath the threshold required for effective signaling, particularly by fast-spiking GABAergic inhibitory neurons and Purkinje cells [1], network instability accompanied by neuroinflammation and ferroptosis may lead to seizure. Consistent with this speculation, lipid biomarkers derived from 15-LO enzyme activity, including 15-HETE, malondialdehyde, and 4-hydroxynonenal, have been proposed to be candidate clinical biomarkers associated with mitochondrial disease and seizure disorders [60–62].

Epilepsy due to mitochondrial defects has been reported to be refractory to current treatment in the vast majority of cases, demonstrating an unmet medical need and underscoring the need for novel therapeutic approaches arising from a nuanced understanding of seizure origins [1,4,25,63], perhaps including those targeting ferroptosis.

### EPI-743 is a first-in-class anti-ferroptotic drug targeting 15-LO

To elucidate the activity and mechanism-of-action of EPI-743, we employed an *in vitro* approach employing enzymology and cell assays with primary patient-derived fibroblasts and B-lymphocytes. Human patient-derived fibroblasts are a widely-used translational model for mitochondrial disease-related epilepsy [28,64], but are just beginning to be used to study ferroptosis/oxytosis [65,66]. We demonstrated that dosed EPI-743 was metabolized in patient-derived fibroblasts to its reduced hydroquinone form (Fig 3A), which dose-dependently inhibited 15-LO in purified enzyme assays (Fig 3B) and decreased levels of the RSL3-induced 15-LO product 15-HETE, but not the 12-LO product 12-HETE, in RSL3-treated cells (Fig 3C and 3D). EPI-743 potently prevented RSL3-induced general lipid oxidation (Figs 1E and 5B; Tables 2 and 3) and ferroptotic cell death induced by two independent challenge conditions in cells derived from multiple patients (Figs 1D, 2B, 5A and 5C; Tables 2 and 3). While the radical trapping activity of EPI-743 has not been reported and hence this mechanism cannot be excluded, our results are consistent with the model that EPI-743 mitigates ferroptosis of PCH6 patient-derived fibroblasts by inhibition of 15-LO by its hydroquinone metabolite.

### Rationale for the clinical evaluation of EPI-743 in pediatric mitochondrial disorders associated with epilepsy

A recent estimate suggests that up to 90% of pediatric epilepsies are refractory to treatment [67]. The majority of currently-marketed anti-epileptic drugs target Na^+^, Ca^2+^, and/or GABA_A_ channel function, but can induce oxidative stress and exacerbate mitochondrial dysfunction. This perhaps explains their lack of efficacy or contraindication in mitochondrial epilepsy patients [4,63,67–69]. We propose that a variety of mitochondrial disease-associated seizure disorders, including Leigh syndrome (*MT-ATP6*, *MT-ND3*, *MT-ND5*, *SURF1*, others), Alpers syndrome (*POLG*), mitochondrial tRNA synthetase syndromes (*RARS2* and others), Rett syndrome (*MECP2*), and *CDKL5* disorder, have different genetic lesions but share common underlying cellular pathologies, namely oxidative stress and ferroptosis. Given the limitations of current therapeutic options, EPI-743 therefore offers a novel target and mechanism for the treatment of pediatric mitochondrial diseases associated with epilepsy.

Indeed, EPI-743 treatment has been shown to reduce the seizure incidence in a small cohort of PCH6 patients [39]. The results presented here support a mechanistic rationale for the therapeutic approach of targeting 15-LO and ferroptosis for the treatment of additional mitochondrial disease patients with epilepsy [39,70].

## Supporting information

S1 Fig RSL3 sensitivity of PCH6 and Leigh Syndrome patient-derived fibroblasts compared to a panel of pediatric healthy control fibroblasts. (A) PCH6 (N = 3 donors) and (B) Leigh Syndrome (N = 3 donors) patient-derived fibroblasts show increased maximal RSL3-induced cytotoxicity when compared to a panel of pediatric apparently healthy control fibroblasts (N = 7 donors). Cell viability was assessed 18h after RSL3 treatment using CellTiter-Glo 2.0 reagent to quantify cellular ATP. Means and best-fit curves (solid lines) from 4-parameter fits with associated 95% confidence bands (dotted lines) displayed. (C) Summary of 4-parameter curve-fit values as calculated in Prism 7.03.
(TIF)

S2 Fig Sensitivity of mitochondrial disease patient-derived fibroblasts to Iron/BSO challenge compared to a reference pediatric healthy control fibroblast. Each pediatric mitochondrial disease patient-derived fibroblast tested showed heightened sensitivity to combination treatment with iron(III) citrate (FeC) and buthionine sulfoximine (BSO) evaluated in parallel to a reference pediatric apparently healthy control fibroblast (GM00038). (A) Subject 070718; Alpers-Huttenlocher syndrome with confirmed *POLG* mutation. (B) Subject GM17567; Rett syndrome with confirmed *MECP2* mutation. (C) Subject 5; Alpers-Huttenlocher syndrome with confirmed *POLG* mutation. (D) Subjects GM01503 and GM03672; Leigh syndrome, mutations not reported. (E) Subject 4; Leigh syndrome due to confirmed *MT-ND3* mutation. Each culture was exposed to a matrix of 4–5 different concentrations each of FeC and BSO. Cell viability by Calcein AM staining was quantified 36-48h after BSO addition and expressed relative to wells in which no FeC or BSO had been added. Mean±SEM (n = 3 replicates) displayed for selected BSO concentrations at which the greatest differential sensitivity compared to GM00038 controls was observed. We note that sensitivity of the GM00038 cells to FeC/BSO challenge is affected by FBS lot and cell passage, contributing to the inter-assay variation observed.
(TIF)

S3 Fig EPI-743 rescue of mitochondrial disease patient-derived cells subjected to ferroptotic challenges. (A) EPI-743 rescue of Rett syndrome fibroblasts (Subject GM17567) treated with 2 μM RSL3 for 24h. Mean±SD (n = 2 replicates) displayed. (B) EPI-743 rescue of EIEE2 syndrome B-lymphocytes (Subject GM23710) treated with 2 μM RSL3 for 48h. Mean±SD (n = 3 replicates) displayed. (C) EPI-743 rescue of EIEE2 syndrome B-lymphocytes (Subject GM23710) challenged with 500 μM FeC and 100 μM BSO for 48h. Mean±SD (n = 3 replicates) displayed. In all panels, cell viability was assessed using CellTiter-Glo 2.0 reagent to quantify cellular ATP.
(TIF)

S1 Table siRNA knockdown of *ALOX15* in PCH6 patient-derived fibroblasts. Summary of Area Under the Curve (AUC) analysis and statistics for the *ALOX15* knockdown data in Fig 4, showing that siALOX15 decreased the sensitivity of PCH6 fibroblasts to a cytotoxic RSL3 challenge, and partially decreased RSL3-induced BODIPY 581/591 C11 lipid oxidation. For each Subject, RSL3 potency AUC values are presented as Total Area and associated Standard Errors, and compared by unpaired t-test. Analysis was performed in GraphPad Prism 8.0.2.
(PDF)

## Abbreviations

ETC: electron transport chain
mtDNA: mitochondrial deoxyribonucleic acid
GSH: glutathione
*GPX4*: glutathione peroxidase 4
PUFA: polyunsaturated fatty acid
15-LO: 15-lipoxygenase (EC 1.13.11.33)
PEBP1: phosphatidylethanolamine binding protein 1
PV: parvalbumin
PCH6: pontocerebellar hypoplasia type 6
OMIM: Online Mendelian Inheritance in Man
EPI-743: vatiquinone / α-tocotrienol quinone
*RARS2*: arginyl-tRNA synthetase 2, mitochondrial
EC: effective concentration
IC: inhibitory concentration
BSO: buthionine sulfoximine
FeC: iron(III) citrate
15-HETE: 15-hydroxyeicosatetraenoic acid
*ALOX15*: Arachidonate 15-Lipoxygenase
LC-MS/MS: liquid chromatography—mass spectrometry
EIEE2: early infantile epileptic encephalopathy type 2
*CDKL5*: cyclin dependent kinase like 5
*MT-ND3*: mitochondrially encoded NADH:ubiquinone oxidoreductase core subunit 3
*POLG*: DNA polymerase gamma, catalytic subunit
*MECP2*: methyl-CpG binding protein 2
ATP: adenosine triphosphate
ROS: reactive oxygen species
GABA: gamma-aminobutyric acid
tRNA: transfer ribonucleic acid
*Trsp*: transfer RNA selenocysteine/phosphoserine (opal suppressor), transfer RNA-SeC (TCA) 1–1
PCH2D: pontocerebellar hypoplasia type 2D
*SEPSECS*: Sep (O-phosphoserine) tRNA:Sec (selenocysteine) tRNA synthase
15-HpETE: 15-hydroperoxyeicosatetraenoic acid
*TLR4*: toll like receptor 4
*MT-ATP6*: mitochondrially encoded ATP synthase membrane subunit 6
*MT-ND5*: mitochondrially encoded NADH:ubiquinone oxidoreductase core subunit 5
*SURF1*: SURF1 cytochrome c oxidase assembly factor
