# Supplementary figures and images for: Targeting ferroptosis: A novel therapeutic strategy for the treatment of mitochondrial disease-related epilepsy
Source: PLoS One. 2019 Mar 28;14(3):e0214250. doi: 10.1371/journal.pone.0214250 (PMC6438538; doi:10.1371/journal.pone.0214250)

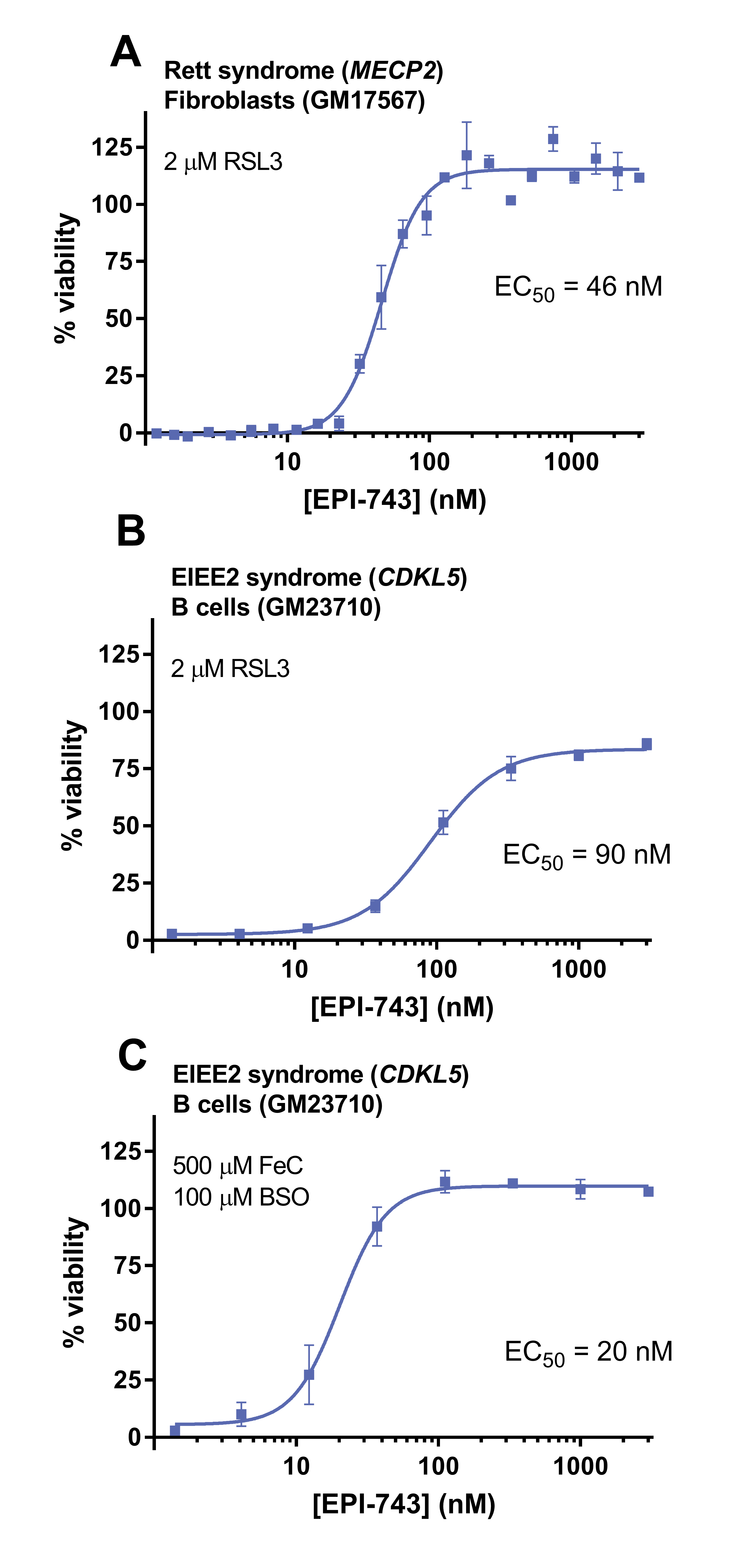

Supplement: S3 Fig — (A) EPI-743 rescue of Rett syndrome fibroblasts (Subject GM17567) treated with 2 μM RSL3 for 24h. Mean±SD (n = 2 replicates) displayed. (B) EPI-743 rescue of EIEE2 syndrome B-lymphocytes (Subject GM23710) treated with 2 μM RSL3 for 48h. Mean±SD (n = 3 replicates) displayed. (C) EPI-743 rescue of EIEE2 syndrome B-lymphocytes (Subject GM23710) challenged with 500 μM FeC and 100 μM BSO for 48h. Mean±SD (n = 3 replicates) displayed. In all panels, cell viability was assessed using CellTiter-Glo 2.0 reagent to quantify cellular ATP. (TIF) [file pone.0214250.s003.tif]
