# Supplementary material for: Targeting ferroptosis: A novel therapeutic strategy for the treatment of mitochondrial disease-related epilepsy
Source: PLoS One. 2019 Mar 28;14(3):e0214250. doi: 10.1371/journal.pone.0214250 (PMC6438538; doi:10.1371/journal.pone.0214250)
Supplement: S1 Table — Summary of Area Under the Curve (AUC) analysis and statistics for the ALOX15 knockdown data in Fig 4, showing that siALOX15 decreased the sensitivity of PCH6 fibroblasts to a cytotoxic RSL3 challenge, and partially decreased RSL3-induced BODIPY 581/591 C11 lipid oxidation. For each Subject, RSL3 potency AUC values are presented as Total Area and associated Standard Errors, and compared by unpaired t-test. Analysis was performed in GraphPad Prism 8.0.2. (PDF) [file pone.0214250.s004.pdf]

SUBJECT 1

	siRNA knockdown effects on RSL3 potency			siRNA knock down effects on RSL-3 induced lipid peroxidation				
				60 nM RSL3		*167 nM RSL3*		
siRNA	*Area Under the Curve (SE)*	*p-value, t-test*	*N*	*Area Under the Curve (SE)*	*p-value, t-test*	*Area Under the Curve (SE)*	*p-value, t-test*	*N*
siControl	238.7 (3.135)		3	49.89 (5.91)		177.4 (5.38)		3
siALOX15	262.5 (3.957)	0.0092	3	15.49 (1.17)	0.0047	144.6 (1.96)	0.0046	3

SUBJECT 2

	siRNA knockdown effects on RSL3 potency			siRNA knock down effects on RSL-3 induced lipid peroxidation				
				60 nM RSL3		*167 nM RSL3*		
siRNA	*Area Under the Curve (SE)*	*p-value, t-test*	*N*	*Area Under the Curve (SE)*	*p-value, t-test*	*Area Under the Curve (SE)*	*p-value, t-test*	*N*
siControl	232.4 (4.733)		3	66.62 (6.30)		148.1 (14.43)		3
siALOX15	285.5 (4.650)	0.0013	3	25.49 (2.55)	0.0038	115.1 (3.54)	0.0905	3

SUBJECT 3

	siRNA knockdown effects on RSL3 potency			siRNA knock down effects on RSL-3 induced lipid peroxidation				
				60 nM RSL3		*167 nM RSL3*		
siRNA	*Area Under the Curve (SE)*	*p-value, t-test*	*N*	*Area Under the Curve (SE)*	*p-value, t-test*	*Area Under the Curve (SE)*	*p-value, t-test*	*N*
siControl	225.7 (5.884)		3	59.11 (1.57)		153.6 (8.57)		3
siALOX15	299.6 (6.298)	0.0010	3	19.1 (1.55)	<0.0001	107.1 (4.75)	0.0090	3
